# Potential Role of Epigenetic Mechanism in Manganese Induced Neurotoxicity

**DOI:** 10.1155/2016/2548792

**Published:** 2016-05-26

**Authors:** Prashant Tarale, Tapan Chakrabarti, Saravanadevi Sivanesan, Pravin Naoghare, Amit Bafana, Kannan Krishnamurthi

**Affiliations:** ^1^Environmental Health Division, CSIR-National Environmental Engineering Research Institute, Nagpur 440020, India; ^2^Visvesvaraya National Institute of Technology (VNIT), Nagpur 440010, India

## Abstract

Manganese is a vital nutrient and is maintained at an optimal level (2.5–5 mg/day) in human body. Chronic exposure to manganese is associated with neurotoxicity and correlated with the development of various neurological disorders such as Parkinson's disease. Oxidative stress mediated apoptotic cell death has been well established mechanism in manganese induced toxicity. Oxidative stress has a potential to alter the epigenetic mechanism of gene regulation. Epigenetic insight of manganese neurotoxicity in context of its correlation with the development of parkinsonism is poorly understood. Parkinson's disease is characterized by the *α*-synuclein aggregation in the form of Lewy bodies in neuronal cells. Recent findings illustrate that manganese can cause overexpression of *α*-synuclein. *α*-Synuclein acts epigenetically via interaction with histone proteins in regulating apoptosis. *α*-Synuclein also causes global DNA hypomethylation through sequestration of DNA methyltransferase in cytoplasm. An individual genetic difference may also have an influence on epigenetic susceptibility to manganese neurotoxicity and the development of Parkinson's disease. This review presents the current state of findings in relation to role of epigenetic mechanism in manganese induced neurotoxicity, with a special emphasis on the development of Parkinson's disease.

## 1. Introduction

Manganese (Mn) is an essential trace element maintained at an optimal level in human body for proper functioning of brain. Manganese also plays significant role in carbohydrate, lipid, and protein metabolism and acts as a cofactor for various antioxidant enzymes like superoxide dismutase, glutamine synthetase, and so forth [1]. Chronic exposure to manganese is known to induce neurotoxicity [2]. Most of the epidemiological studies on exposure to manganese pertain to its effect on occupational workers. High environment level of manganese may be a potential risk factor in causing disorder associated with developing nervous system as well as for adult brain [3, 4]. Welders and miners are the diverse occupational population who are exposed to manganese. Manganese, through inhalation, enters the body circulation and affects the central nervous system [5]. The neurological disorders similar to schizophrenia and Parkinson's disease (PD) have been observed to be due to excessive manganese intake from both environment and occupational settings characterized by dopaminergic cell death [6, 7]. Occupational exposure of manganese at an elevated level may cause manganism with neurological symptoms resembling idiopathic Parkinson's disease (IPD) [8]. Settivari's study showed the probable genetic linkage between manganism and idiopathic Parkinson's disease (IPD) [8]. This suggests the common molecular modalities involved in pathophysiology of both disorders. However the identification of key molecular determinants largely remains elusive. This prolonged exposure has been associated with increased risk for PD [9, 10]. Initiation of oxidative stress resulting from discrepancy between ROS and antioxidant generation is considered to be the probable mechanism of neurotoxicity as manganese can initiate apoptosis and/or necrotic cell death in neuronal or glial cells [11–13].

Dynamic chromatin remodeling governs the accessibility of transcription factors to promoters regions of genes, thereby regulating gene expression [14]. Epigenetic mechanisms such as DNA methylation, histone modification, and microRNAs participate in regulating the gene expression. A growing number of evidences suggest that exposure to environmental toxicants may induce alterations in gene expression through epigenetic mechanisms that can be linked to disease susceptibility and development. Epigenetic deregulation may lead to the development of neurological disorders like Parkinson's disease, Huntington's disease, and mood disorders (depression and anxiety) [15]. Millions of people worldwide are affected by the most common movement disorder known as Parkinson's disease (PD). Apart from mutation in genome, various environmental and occupational factors such as pesticides like paraquat and metals such as manganese have been considered as risk factor for the development of Parkinson's disease [16]. Parkinson's disease is characterized by the presence of *α*-synuclein proteinaceous inclusions, known as Lewy bodies (LBs) and Lewy neurites (LNs) in the dopaminergic neurons of brain. *α*-Synuclein aggregation in neuronal cells in form of Lewy bodies is a pathological hallmark of Parkinson's disease. Manganese has been found to accelerate the *α*-synuclein aggregation in neuronal cell line by inducing the overexpression of *α*-synuclein [17]. The significance of elevated *α*-synuclein expression can be understood by the fact that 50% increase in *α*-synuclein expression has been hypothesized to cause Parkinson's disease [18]. ROS have the potential to influence epigenetic processes through oxidative stress mechanism [19].

The review is an effort to generate the working hypothesis based on the present knowledge of mechanism underlying the manganese exposure, parkinsonism, and epigenetic modifications over genetic susceptibility. There are many assumptions suggested, lacking epigenetic studies on manganese exposure. It is also an attempt to link the possible role of epigenetic mechanism in implication of Parkinson's disease induced by manganese. The hypothesis indicated in this review may be relevant and needed consideration in future studies.

## 2. Manganese in Environment and Its Route of Exposure

Even though manganese is required in trace amount, exposure from both environmental and occupational settings has been reported to be toxic for brain and central nervous system (CNS). During each life stage the increased exposure to manganese may occur with different absorption routes. Hence lifetime exposure to manganese is important in considering its toxicological aspect [20].  Table 1 depicts the major sources and route of manganese exposure. Relative potential for exposure depends on the sources of exposure. For example, intake due to inhalation of ambient air has higher risk potential compared to that of the ingestion of manganese from diet, drinking water, and so forth.

Manganese is common constituent of ground water. Elevated level of manganese may exist because of certain bedrock formation (geogenic) or anthropogenic activities [21]. Common population could be exposed to high level of manganese through consumption of well/drinking water or inhalation of atmospheric air with manganese released as a combustion product of methylcyclopentadienyl manganese tricarbonyl (MMT) (a petrol antiknock additive) [21–23]. Exposure to manganese through contaminated drinking water that exceeds the WHO regulatory standards (400 *μ*g/L) has been associated with adverse neurological problems [24, 25]. Manganese exposure through water rather than diet has been associated with significant deposition of manganese in hair samples of children [26]. Manganese has been found in breast milk of population residing near to industrial steel plant [27]. As a nutritional supplement, manganese is added to a variety of foods such as soy containing infant formulas. The highest concentration of manganese has been reported in herbal food including grain, rice, nuts, and tea [28–30]. Manganese-containing pesticides maneb and paraquat have been used to treat various plant pathologies [31]. An epidemiological observation has reported an increased risk of developing Parkinson's disease in rural area population exposed to these pesticides [31–33]. Elevated intake above a recommended level may be a concern in dietary foods for general population. Neurotoxicity of manganese varies with its route of exposure. Toxicity of manganese is least among all other toxic trace metals when uptake is via ingestion compared to its exposure through inhalation (Table 1). Inhaled manganese provides a direct path to brain tissue through olfactory neural pathway (nasal airways) while lung uptake has long residence time with continuous source of exposure. The intake of manganese via inhalation rather than ingestion is a major concern as it is evident that inhaled manganese enters the body circulation and can be directly transported to the central nervous system [34]. Manganese is found at higher level in ambient air near industries engaged with processing or use of manganese. To increase the strength and stiffness, manganese is used in the steelmaking industries. The miners and workers of ferroalloy, smelting, and metallurgy industries along with battery and car machine manufactures are the heterogeneous population who are occupationally exposed to respirable metal dust particles [35–37]. The Unified Parkinson's Disease Rating Motor Scale 3 (UPDRS3) was used for accessing the risk of parkinsonism in welding fumes exposed workers. The results of the study depict significant difference in the UPDRS3 scale of welding exposed and nonwelder reference subjects [38]. This signifies the association between welding fume exposure and parkinsonism. Positron emission tomography and radiotracer FDOPA based investigation of welding workers with mild parkinsonism sign have shown the evidence of dopaminergic dysfunction in the caudate nucleus [39]. This strengthens the evidence for neurotoxic effects of welding fumes.

**Figure 1:**
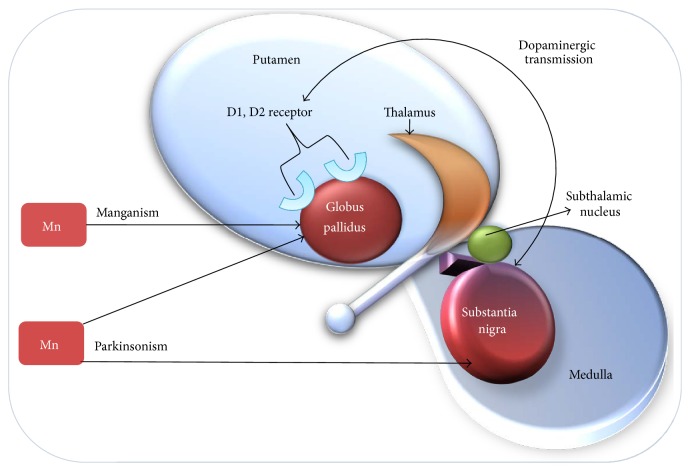
The specific regions of the brain influenced via manganese exposure depending on the level and potential of exposure. Globus pallidus is a major part of basal ganglia region of brain that includes substantia nigra. Dopamine is an important neurotransmitter, produced in the brain mainly by dopaminergic neurons in substantia nigra. The progressive loss of dopaminergic neurons due to manganese accumulation in substantia nigra over the prolonged exposure period leads to development of parkinsonism. Manganism happens on high acute exposure and accumulation of manganese, specifically in the globus pallidus.

Epidemiological observation and studies show that manganism and parkinsonism are the two manifestations of manganese exposure. These neurodegenerative manifestations may stand at two extreme conditions depending on the intensity, duration, and route of exposure [40]. Manganism is the result of high acute exposure whereas parkinsonism happens due to low chronic exposure. According to the WHO, manganism can occur when the airborne concentration of manganese in inhalable particles is above 1 mg/m^3^ while parkinsonism may result from lifetime exposure to much lower concentration of around 100 ng/m^3^ of manganese in respirable dust particles [35, 41–43]. The delayed and long term toxicity may result due to cumulative exposure to manganese along with other known neurotoxicants such as pesticide and lead. Brain may be unable to compensate the exposure to multiple neurotoxicants leading to persistent and cumulative damage [44]. Thus, lifetime chronic exposure to manganese from prenatal to older may be considered as a risk factor for development of parkinsonism [42]. Morphological, neurochemical, and behavioral alterations were reported in rat exposed to MnCl_2_/Mn(OAc)_3_ through inhalation route. These alterations were similar to those observed in PD [45]. Entering the brain, manganese gets accumulated in several regions particularly in globus pallidus, substantia nigra pars compacta, and other ganglia structures [46]. Manganism appears due to accumulation of manganese specifically in globus pallidus region of brain, whereas parkinsonism results from accumulation in other brain regions including globus pallidus and substantia nigra as depicted in Figure 1. Although globus pallidus is major site of Mn deposition in manganism but recent evidence suggests that Mn deposition may also occur in substantia nigra [47–49]. Substantia nigra is a specific brain region concerned with dopamine production. The progressive loss of dopaminergic neurons on manganese accumulation in this region is responsible for the pathophysiology of parkinsonism. PET neuroimaging studies carried out on animal models chronically exposed to Mn reported movement abnormalities in absence of nigrostriatal neuronal degeneration through inhibition of striatal dopamine release [50–52]. These studies revealed dysfunction of dopaminergic system in the cause of motor deficit. However, the detailed mechanism regarding whether Mn could have an impact on motor abnormalities in absence of dopaminergic neurodegeneration needs to addressed using advance molecular biology tools. The magnetic resonance imaging (MRI) of parkinsonism patients with history of exposure to manganese indicated the increased intensity in globus pallidum and substantia nigra regions of brain [53]. T1-weighted magnetic resonance imaging (MRI) and fluorodopa positron emission tomography (PET) scans of patients diagnosed with idiopathic parkinsonism, including the career welders, were carried out [54]. The results of the scans were found to be typical of idiopathic parkinsonism and were similar for both welder and nonwelder populations. Moreover the 6-(18F) fluorodopa PET scan of 43-year-old female parkinsonism patient was found to be abnormal while T1-weighted magnetic resonance imaging (MRI) of internal segment of the globus pallidum displayed increased signal [55]. These studies concluded that the manganese induced parkinsonism and Parkinson's disease share similar clinical and pathophysiological features.

## 3. Oxidative Stress Mechanism in Manganese Neurotoxicity

**Figure 2:**
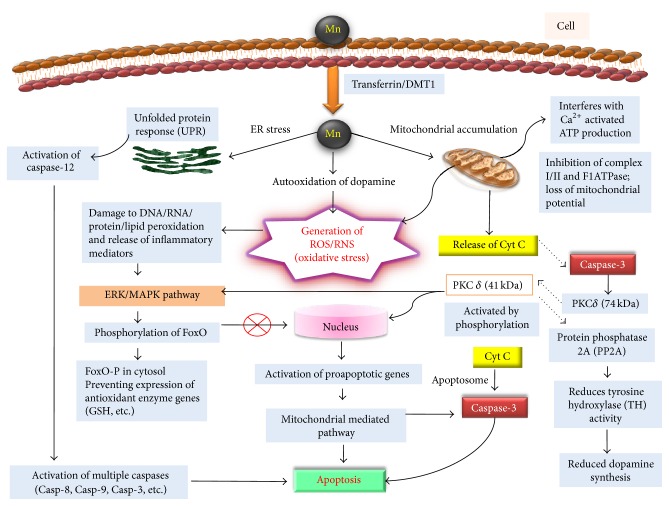
Schematic summarizing the oxidative stress mediated apoptotic pathway through which manganese causes the dopaminergic cell death and is involved in neurotoxic effects. Mn enters neuronal cells via transferrin/DMT1 receptor. Inside cell, Mn primarily gets accumulated in mitochondria causing inhibition of complex I/II and F1ATPase and interferes with Ca^2+^ activated ATP production. This resulted in loss of mitochondrial potential with release of Cyt C. Mitochondrial dysfunction also causes the generation of oxidative stress (ROS/RNS). The oxidative stress resulting from dopamine autooxidation causes damage to DNA/RNA/protein/lipid and releases inflammatory mediators that activate ERK/MAPK pathway. Activated kinase phosphorylates FoxO, preventing its translocation to nucleus and activation of antioxidant enzyme genes. Mn may also cause the endoplasmic reticulum (ER) stress that resulted in activation of caspase-12 through unfolded protein response. Caspase-12 may further activate the other downstream multiple caspases which culminates in apoptotic cell death. Cyt C released upon mitochondrial damage activates caspase-3, a critical regulator of apoptosis. Caspase-3 proteolytically activates the protein kinase C *δ* (PKC*δ*) that again can activate ERK/MAPK pathway or on translocation to nucleus causes the expression of proapoptotic genes, resulting in mitochondrial mediated apoptotic cell death. Protein phosphatase 2A is a downstream substrate for PKC*δ*; activation of PP2A reduces the activity of tyrosine hydroxylase, inhibiting the dopamine production.

Manganese induced oxidative stress is known to cause dopaminergic cell death via apoptosis (Figure 2). The transport of manganese to cellular system occurs by simple diffusion processes. Other factors like diet such as body iron status may also determine the absorption of manganese [56, 57]. There is an inverse relationship between the body iron status and manganese uptake since both compete for the same transport machineries such as transferrin/divalent metal transporter-1 (DMT1) [34, 57]. Various* in vivo* and* in vitro* cell models (representing central nervous system and brain) have been used to gain the mechanistic insight of manganese neurotoxicity. All these studies have identified the common signaling molecules such as PKC*δ* that leads to apoptosis in these cellular systems [58]. Upon entering the cell Mn may have three cellular targets as shown in Figure 2. It may induce the endoplasmic reticulum stress and/or can accumulate in mitochondria and/or can cause the dopamine autooxidation. Mitochondria are an important cellular target in manganese neurotoxicity. Mitochondrial accumulation of manganese results in the inhibition of oxidative phosphorylation and generation of reactive oxygen species (ROS) [59]. Mn was found to interact with complex I and/or complex II and F1ATPase and may also interfere with Ca^2+^ activated ATP production upon mitochondrial accumulation (Figure 2) [60–62]. The intramitochondrial Ca^2+^ regulates the rate of ATP production via binding a series of Ca^2+^-sensitive sites. Ca^2+^ activated dehydrogenases are associated with the Krebs cycle such as pyruvate dehydrogenase (PDH), isocitrate dehydrogenase (ICDH), *α*-ketoglutarate dehydrogenase (*α*KGDH), and F1F0 ATP synthase [63]. Mitochondrial accumulation of manganese was found to interfere with the Ca^2+^ activated ATP production through binding with Ca^2+^-sensitive sites in mitochondrial metabolic enzymes with more affinity than that of Ca^2+^ itself [64]. ATP is an important paracrine signaling molecule mediating the intracellular communication between the astrocytes through transmission of Ca^2+^ waves. Ca^2+^ signaling is a critical process for regulating the synaptic function, metabolism, and cerebral blood flow in central nervous system [65]. The neurodegenerative diseases such as Parkinson's and Alzheimer's diseases have been associated with dysfunction in the Ca^2+^ signaling [66]. Transient receptor potential channel TRPC3 was linked with pathophysiological activation of astrocytes [67]. Recently the ability of manganese to affect Ca^2+^ signaling was studied in striatal astrocytes and Mn was found to inhibit the Ca^2+^ influx in astrocytes through receptor potential channel TRPC3, most likely through competition for Ca^2+^ influx through these cation channels (Figure 2) [68]. However, more detailed electrophysiological studies are needed to precisely predict the mechanism through which manganese induced disruption of Ca^2+^ signaling in astrocytes.

Exposure to manganese causes the release of cytochrome C from mitochondria followed by subsequent loss of mitochondrial electrical potential (Figure 2). Cytochrome C can initiate a chain of molecular signaling events leading to caspase-3 mediated apoptosis. Caspase-3 is a key mediator of apoptosis and presumed to be activated downstream to cytochrome C. Protein kinase C (PKC*δ*) has been identified as a proximal regulator of apoptosis [58]. Critical regulation of its cleavage and transportation to nucleus may determine the fate of cell survival or cell death. The nuclear transport of its activated form of cleaved product (41 kDa) makes the cell irreversibly committed to apoptosis. The proapoptotic PKC*δ* (74 kDa) is reported as a downstream substrate for caspase-3 released from mitochondria as a result of mitochondrial damage (Figure 2). Caspase-3 cleaves the PKC*δ* (74 kDa) to its proteolytic active product PKC*δ* (41 kDa) that can translocate to nucleus and may cause the expression of proapoptotic genes [58, 69]. Activation of PKC*δ* has been identified as a key event in the dopaminergic degeneration in animal PD models and apoptotic cell death of dopaminergic neuronal cells (N27) exposed to 6-hydroxydopamine (6-OHDA) [70]. Oxidative stress induced mitochondrial dysfunction upon 6-OHDA exposure followed by release of caspase-3 considered to be responsible for proteolytic activation of PKC*δ* [70].

Protein phosphatase 2A (PP2A) is known to regulate the activity of tyrosine hydroxylase (TH) enzyme which is responsible for dopamine synthesis [71]. Recently both TH and PP2A were found to interact and colocalize with PKC*δ*. PKC*δ* may then phosphorylate PP2A to enhance its phosphatase activity. Increased PP2A activity dephosphorylates TH-ser40 that reduces its enzymatic activity, thereby depleting dopamine in nitrostriatal neurons as a result of manganese exposure (Figure 2) [71]. The dopamine depletion is dependent on the concentration and duration of manganese exposure [72]. Recently zebrafish model of manganism was reported to decrease tyrosine hydroxylase in catecholaminergic nuclei of Mn exposed zebrafish larvae [73]. These results are in agreement with previous* in vitro* studies. There has been a functional biphasic variation in TH activity depending on chronic or acute level of Mn exposure. Chronic exposure leads to decrease in TH activity while acute exposure causes increase in the TH activity. The biphasic variation in TH activity has also been reported in human Mn studies. Although the cause of biphasic variation is not known, it may be hypothesized to be related to U shaped relations underlying deficiency and toxicity conditions, governed by dose and duration of exposure. Animal models may be used to investigate the detailed mechanism responsible for the cause of biphasic variation in TH activity. Inhibiting the PKC*δ* function was found to restore TH and PP2A activities to normal, suggesting involvement of PKC*δ*-PP2A signaling pathway in manganese induced reduction in TH activity [74, 75]. Manganese ions are required for PP2A phosphatase activity [76]. The release of Mn from PP2A active site by PP2A specific methylesterase leads to its inactivation.

Manganese has been reported to alter the catabolism of dopamine in dopaminergic cells [42, 77]. This causes the autooxidation of dopamine, contributing to the generation of reactive oxygen/nitrogen species ROS/RNS (Figure 2) [77, 78]. Free radicals cause structural and functional modifications of proteins DNA and RNA along with lipid peroxidation (F2-isoprostanes) [62, 79, 80]. Generation of free radicals causes release of inflammatory mediators like prostaglandin contributing to apoptotic cell death [81–83]. The pathways that resulted in oxidative stress, inflammation, and apoptosis upon Mn exposure are linked with each other and are responsible for the pathophysiology of neurodegenerative diseases [84]. MAPK kinase (ERK, JNK, and p38 MAPK) regulates a variety of cellular processes such as differentiation, proliferation, and inflammatory response by acting as connecting link between the extracellular signals and intracellular signaling pathway through intermediate signaling molecules [85]. ROS generated as a result of mitochondrial respiratory dysfunction on manganese exposure has been reported to trigger the activation of ERK and p38-MAPK analogous to that of ultraviolet radiation induced ROS generation and MAPK activation (Figure 2) [86–88]. Dopamine producing cells exposed to manganese has been reported to cause reduction in glutathione and glutathione peroxidase synthesis, an important antioxidant in cellular defense against oxidative stress [3, 68, 89]. SHSY5Y cells exposed to manganese were reported to cause oxidative DNA damage. These cells were found to be rescued when supplemented with antioxidants N-acetyl cysteine and glutathione. This suggests the involvement of ROS and depletion of antioxidants in apoptotic cell death. Expression of antioxidant genes is tightly regulated inside the cell. A forkhead box transcription factor class O (FoxO) is a critical transcriptional factor that regulates these antioxidant genes [90]. Phosphorylation and dephosphorylation events of FoxO decide the fate of its activity. Dephosphorylated form is located in nucleus where it mediates the transcription of antioxidant/protective genes on manganese exposure. Recently FoxO has been reported as one of the downstream substrates for MAPK kinase in rat neonatal astrocyte cultures (Figure 2) [90]. Increased phosphorylation of FoxO prevents its translocation to cell nucleus, thus inhibiting the transcription of antioxidant genes contributing as one of the factors to the chain of events which results in neuronal cell death [91]. Recently* C. elegans* models of manganism have reported that the loss of glutathione S-transferase (GST-1) associated with increased loss of dopaminergic neurons and neurodegeneration [8]. The other downstream substrate for PKC*δ* mediated apoptotic cell death has not been yet identified but it has been proposed that factors such as DNA protein kinase (DNA-PK), MAP-kinase, scramblase, and NF-kappa transcription factor may be involved in this process [92–94].

Manganese exposure was found to cause endoplasmic reticulum (ER) stress in nigral dopaminergic neuronal cells SN4741. The ER stress results in the upregulation of unfolded protein response genes such as BiP and activation of caspase-12 (Figure 2) [95]. Caspase-12 subsequently activates multiple downstream caspases (Casp-8, Casp-9, Casp-1, and Casp-3) which leads to apoptosis in SN4741 cells. Manganese exposed T98G cells have shown significant upregulation of caspase-3 in proportion to that of elevated DNA fragmentation [96]. The mechanism that causes ER stress and caspase-12 mediated activation of multiple caspases in response to manganese exposure is not being studied. However overexpression of Bcl-2 in these cells has been shown to have neuroprotective function that confirms the role of caspases in neuronal cell death.

1-Methyl-4-phenyl-1-2-3-6-tetrahydropyridine (MPTP) is a precursor to 1-methyl-4-phenylpyridinium (MPP^+^), a known neurotoxin responsible for causing the death of dopaminergic neurons in substantia nigra of brain and inducing permanent symptoms of Parkinson's disease [97]. Manganese treated PC12 cells have shown similar effects and mode of action to that observed with MPTP exposure [87]. Majority of* in vitro* studies have focused on the effect of manganese exposure on dopaminergic system while relatively few studies have considered nondopaminergic system [91]. Divalent metal transporter-1 (DMT1) is major transporter protein for manganese and is reported to be present in inner ear cells [98, 99] although the exact cellular location of DMT1 is still not determined. Manganese was reported to be toxic for neurons and sensory cells in cochlear cultures from postnatal rats suggesting that hearing deficit may occur with excess manganese exposure [100].

### 3.1. Oxidative Stress Influencing Epigenetic Regulation

Recent studies have focused on the epigenetic mechanisms that can modify the regulation of gene expression. Epigenetic alterations do not involve the change in nucleotide sequence but rather influence the process that governs gene expression. The epigenetic processes that have been recognized to regulate gene expression are DNA methylation and histone modification along with miRNAs that act at posttranscriptional level. DNA methylation in conjugation with histone modification controls the accessibility of transcription factors to promoter region of genes. There is growing evidence suggesting the implication of epigenetic mechanisms in susceptibility and progression of environmental toxicants induced diseases [101, 102]. Epigenetic mechanism has been also reported to be responsible for causing various neurological disorders [15]. Upon manganese exposure, oxidative stress mediated apoptotic cell death has been well recognized mechanism for neuronal cell death. Generation of oxidative stress and its influence on epigenetic regulation have been investigated in several* in vitro* and* in vivo* models but not in the context with manganese induced neurotoxicity.

Exposure to various heavy metals including As, Pb, Cd, and Ni has been reported to alter DNA methylation levels [103]. Heavy metal exposure generates reactive oxygen species through redox cycling [104]. Inhibition of antioxidant defense system may further potentiate ROS induced oxidative damage as reported and already discussed in relevance to manganese exposure in the previous section. The DNA lesions such as base modifications, deletions, and chromosome rearrangements caused by oxidative stress may interfere with ability of DNA methyltransferase to use DNA as a substrate, altering the global or gene specific methylation [105–107]. Alternations in DNA methylation mostly occur at CpG sites situated at the promoter region of genes. Methyl group in 5-methylcytosine has role in binding to transcriptional factors and transcriptional repressor methyl-CpG binding protein 2 (MeCP2) in sequence specific DNA protein interactions [108–110]. DNA methylation at CpG sites either prevents binding of transcription activators or recruits the transcriptional repressor (MeCP2) that may alter gene expression by interacting with histone deacetylase (HDAC). DNA methylation in association with histone deacetylase mediates repression of gene expression by generating the heterochromatin regions that result in potential heritable epigenetic alterations. 5-Methylcytosine is prone to oxidation and hydroxymethylcytosine is formed as a result of methylation. This reverses the binding of MeCP2 and further steps in chromatin condensation and silencing of gene expression [111]. ROS induced oxidative DNA damage leads to the global hypomethylation at CpG sites [112]. The global DNA methylation level was found to be increased in BEAS-2B cell exposed to 150 *μ*M H_2_O_2_ for 3 days [113]. Additionally, BEAS-2B cells chronically exposed to arsenic were reported to induce ROS and promote tumorigenesis via downregulation of miR-199a-5p [114]. HIF-1*α* and COX-2 are identified as the downstream targets of miR-199a-5p [113]. The activation of HIF-1*α* via epigenetic mechanism was found to be associated with the occurrence of several types of cancer [115, 116]. Hypermethylation of glutathione-S-transferase P1 (GSTP1) gene was reported in intraepithelial neoplasia (HG-PIN) that makes the cells more susceptible to oxidative stress mediated DNA damage [117]. Gene specific promoter methylation in response to oxidative stress has been reported in the tumor suppressor gene RUNX3 [107, 118]. Methylation of E-cadherin promoter was reported in human colorectal and hepatocellular carcinoma cells [119]. Methyl-CpG binding protein 2 (MeCP2) is key contributor in epigenetic silencing of genes. MeCP2 gene mutation has been reported to cause neurodevelopmental disorder (Rett syndrome) [107]. The MeCP2 gene promoters have been found to be heavily hypermethylated in neuronal tissues. These results were confirmed by chromatin immunoprecipitation with anti-MeCP2 antibody in mice neuronal tissue cells [120]. The reactive oxygen species (ROS) generated as a result of iron sulfate or aluminum sulfate exposure have been reported to cause the upregulation of miRNAs, such as miR-9, miR-125b, and miR-128, in* in vitro* cell models [121]. Histone methylations primarily, histone H3 and histone H4, were among the important epigenetic modifications [122]. Recently, oxidative stress generated through occupational exposure to arsenic was reported to increase H3K4me2 leading to transcriptionally active euchromatin regions [123]. This suggests the influence of oxidative stress on epigenetic regulation in cells. The histone deacetylase such as HDAC3 was found to be susceptible to oxidative damage. Thus oxidative stress mediated loss of activity may affect the global histone deacetylation [80]. Recent studies in context with alteration of epigenetic mechanism in response to environmental toxicants entail the contribution of oxidative stress as probable factor in mediating epigenetic changes [123].

These studies firmly confirm the ability of oxidative stress to deregulate the epigenetic mechanism of gene regulation. Oxidative stress mediated mechanism has been well established in manganese induced neurotoxicity; however the potential involvement of epigenetic deregulation has not been addressed. Studies involving the impact of Mn exposure on epigenetic regulation are not available. Hence at present, in-depth investigation is required on the oxidative stress mediated epigenetic alteration upon manganese exposure. This will facilitate better understanding of the potential role of epigenetic mechanism in manganese induced neurotoxicity.

## 4. Epigenetics of Parkinson's Disease

**Figure 3:**
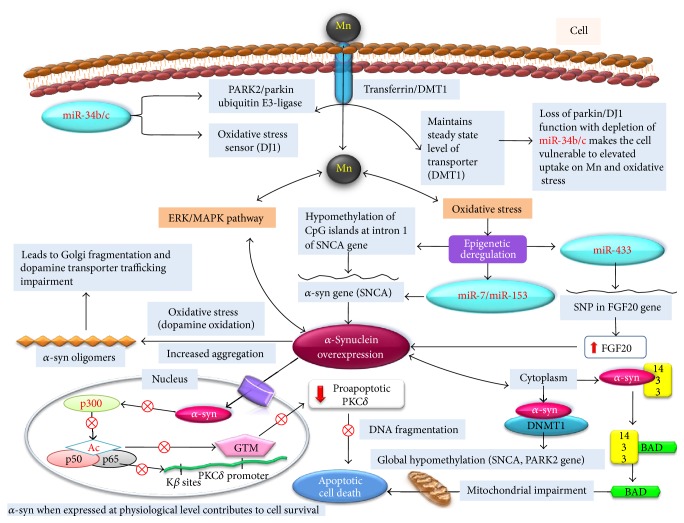
Schematic shows the potential role of epigenetic mechanism that may be involved in manganese induced chronic neurotoxic effects and in pathology of Parkinson's disease. The steady state level of Mn transporter (DMT1) is maintained by PARK2/parkin ubiquitin E3-ligase. miR-34b/c regulates the expression of PARK2. Loss of its expression can make the cells susceptible to elevated uptake of Mn. DJ1 works as an oxidative stress sensor which is again regulated by miR-34b/c; hence deregulation in miR-34b/c expression can make the cell vulnerable to oxidative stress. Mn induced oxidative stress causes the overexpression of *α*-synuclein via ERK/MAPK pathway or through epigenetic alteration in miR-7/miR-153 expression which regulates *α*-syn gene. Oxidative stress may also cause the hypomethylation of CpG islands at intron 1 of SNCA gene resulting in *α*-synuclein overexpression. Expression of *α*-synuclein is regulated by fibroblast growth factor 20 (FGF20) which is in turn regulated by miR-433. The single nucleotide polymorphism (SNP) at binding site of miR-433 disturbs epigenetic regulation of FGF20 causing *α*-synuclein overexpression. *α*-Synuclein under the influence of oxidative stress can form *α*-syn oligomers which leads to Golgi complex fragmentation and subsequent impairment in dopamine transporter biosynthesis and trafficking. *α*-Synuclein at cytoplasm binds to antiapoptotic 14-3-3, making proapoptotic BAD cause mitochondrial impairment. *α*-Synuclein is reported to sequester DNMT1 in cytoplasm which results in global hypomethylation of genes including those associated with Parkinson's disease (SNCA, PARK2, etc.). *α*-Synuclein expressed at physiological level is supposed to participate in cell survival via regulating proapoptotic PKC*δ* expression through epigenetic mechanism. *α*-Synuclein at nucleus binds to histone proteins and inhibits activity of histone acetyltransferase, causing deregulation of PKC*δ* expression. The inhibition of PKC*δ* expression resulted in DNA fragmentation followed by apoptotic cell death.

Parkinson's disease (PD) is amongst the common and well known neurodegenerative diseases mostly characterized by the presence of Lewy bodies in dopaminergic neuronal cells. PD is described as the idiopathic syndrome of parkinsonism. However, studies have correlated the occurrence of PD with the inheritance of defective genetic signatures [124, 125]. Generation of oxidative stress, mitochondrial dysfunction leading to energy imbalance, and defective ubiquitin mediated proteasomal cellular pathways has been identified to be responsible for PD [126]. A recent study entails that environmental pollutant induced epigenetic modifications are capable of bringing the perturbation to gene expression as similar to that observed with pathogenic gene mutations in PD [92]. Long term chronic exposure to certain metals like copper and manganese individually or in combination has been significantly found to be connected with increased risk of Parkinson's disease [9, 127]. At cellular level PD is often characterized by eosinophilic Lewy bodies in dopaminergic neurons of substantia nigra of the brain consisting of aggregates of normal and misfolded proteins [128]. Recently, mutation in parkin gene was reported in PD patients of south India, without the development of Lewy bodies [129]. Clinical symptoms of PD are observed through progressive loss of dopaminergic neurons followed by reduced secretion of dopamine [130]. Mn exposure causes reduced secretion of dopamine through PKC*δ*-PP2A signaling via inhibiting the TH activity. This finding supports the definitive role of environmental Mn exposure in etiology of PD. However review paper by Guilarte raises controversy against the similarities between manganese induced parkinsonism and idiopathic Parkinson's disease [131]. The hypothesis of the review was based on the studies performed on IPD patients to their response to levodopa therapy at different time period during the course of disease. Hence direct correlation between manganese induced parkinsonism and IPD may be problematic. Familial cases of PD associated with genetic mutation in SNCA, parkin, UCHL-1, PINK1, DJ1, and LRKK2 genes contribute only 5–10% of PD cases while the majority of PD cases are idiopathic [132, 133]. Recessive mutation to other PD linked genes such as parkin/PARK2 and DJ1 leads to loss of their functions and is reported to be responsible for early onset of parkinsonism [134]. Parkin is E3 ubiquitin ligase that regulates protein functions via targeting them for proteasomal degradation [134].  Figure 3 entails the molecular (genetic/epigenetic) events involved during the development of PD. Divalent metal transporter DMT1 has broad specificity for transport of metal ions but plays a significant role in transport of manganese [135, 136]. DMT1 is a substrate for parkin and hence is involved in maintaining steady state level of this transporter [137]. Loss of parkin function makes the cell susceptible to elevated uptake of manganese [138]. DJ1 function is an oxidative stress sensor in neuronal cells of brain. The inactive oxidized form of DJ1 was found to be elevated in sporadic PD brain patients [139]. Inactivation of DJ1 can make the cell more vulnerable to the oxidative stress induced neuronal cell death (Figure 3) [140]. Simultaneous miRNA analysis in differentiated SH-SY5Y cell line and PD brain samples revealed depletion or reduction in miR-34b/c [141, 142]. Further, this reduction was found to be associated with reduced parkin and DJ1 protein expression [143]. This suggests the adverse effect of manganese exposure on epigenetic mechanism. Dysregulation in parkin and DJ1 function makes the cell prone to oxidative stress and mitochondrial dysfunction that compromises cell viability as shown in Figure 3 [144, 145]. However, the components of mitochondrial proteins that are modulated by miR-34b/c disruption need to be identified. Related studies have demonstrated that overexpressing parkin and PD linked genes can eliminate the *α*-syn induced neurotoxicity. It was found that E3-ligase activity of parkin is responsible for degradation of *α*-syn [146]. The release of cytochrome C from mitochondria as a result of mitochondrial membrane disruption is crucial for initiation of apoptosis. Recently parkin has been reported to inversely affect the release of cytochrome C and mitochondrial function in SH-SY5Y cells along with murine and fly models of parkin deficiency [145, 147, 148].

Alpha-synuclein (*α*-syn) is small soluble protein (14 kDa) expressed primarily at presynaptic terminals and is natively unfolded, although it gets folded when associated with membrane and when binding lipid molecules [149, 150]. *α*-syn has gathered considerably much interest in recent years as alteration in its gene expression was reported to be involved in the cause of several neurodegenerative diseases including Parkinson's disease [151, 152].* In vitro* investigations have revealed that *α*-syn is a major component of Lewy bodies consisting of aggregates of *α*-syn protofibril formed as a result of excessive oxidative stress generated through autooxidation of neurotransmitter dopamine [153–155]. Alpha-synuclein overexpression can significantly contribute to the increased risk of sporadic PD [156, 157]. PD patient's brain samples have shown the hypomethylation of* SNCA* intron 1 correlating with an increased expression of *α*-syn (Figure 3) [158–162]. Apart from DNA methylation, miRNAs were also reported to regulate the *α*-syn expression at posttranscriptional level. Specifically miR-7 and miR-153 were reported to regulate *α*-syn expression in worm* C. elegans* and PD patients brains (Figure 3) [163, 164]. Additionally, impairment in miR-64, miR-55, let-7, miR-10a, miR-10b, miR-132, miR-7, miR-153, and miR-435 expressions in symptomatic transgenic PD mice and PD disease models was found to be linked with increased *α*-syn level [163, 165–167]. FGF20 is a member of fibroblast growth factor family that regulates the development and function of central nervous system. Variation in miR-433 binding site of FGF20 resulted in overexpression of FGF20 [168]. Elevated FGF20 may be linked with increased *α*-syn expression in PD patient brain (Figure 3) [169].

## 5. Implication of Epigenetic Mechanism in Manganese Induced Parkinson's Disease

*α*-Synuclein has been known to play a significant role in the dopaminergic cell death as observed in Lewy body diseases such as Parkinson's disease [170, 171]. The manganese exposure generates the oxidative stress and causes dopamine oxidation. Oxidative stress was also considered as a critical mediator of dopaminergic cell death in Parkinson's disease [172, 173]. Exposure to manganese has been reported to cause overexpression of *α*-synuclein in neuroblastoma cells (SH-SY5Y) and implicated to be involved in neuronal apoptosis (Figure 3) [18, 174]. However, the upstream mechanism leading to *α*-synuclein overexpression in cells exposed to manganese is still unexplored. A recent finding has demonstrated the involvement of ERK1/ERK2 MAPK pathway responsible for *α*-synuclein overexpression in PC12 cells exposed to manganese as shown in Figure 3 [17]. Hypomethylation of *α*-synuclein gene promoter has been demonstrated to cause overexpression of *α*-synuclein in PD and related disorder [162]. Thus it may be hypothesized that alterations in methylation pattern may be responsible for manganese induced *α*-synuclein overexpression.

Overexpression of *α*-synuclein may lead to the onset of apoptosis (Figure 3). Oxidative stress and neurotransmitter dopamine may contribute to the *α*-synuclein aggregation in* in vitro* models. *α*-Synuclein aggregation may further damage dopaminergic neurons in worm* C. elegans* [151, 175, 176]. *α*-Synuclein aggregation was reported to impair the biosynthetic pathway for dopamine transporter biosynthesis and subsequent maturation and trafficking (Figure 3) [177]. *α*-syn may form the oligomers under the influence of oxidative stress that may lead to the fragmentation of Golgi complex [177].

*α*-Synuclein has been recognized to play dual role in neuronal cell. When expressed at normal physiological level *α*-synuclein is neuroprotective while overexpression beyond a certain threshold level resulted in toxicity (Figure 3) [178]. The nuclear localization of *α*-synuclein was reported to cause the downregulation of proapoptotic PKC*δ* (Figure 3) [179].

Oxidative stress was identified to be responsible for the nuclear translocation of *α*-synuclein in dopaminergic cells [180]. Nuclear translocation of *α*-synuclein has been shown to cause increased susceptibility to oxidative stress in MES23.5 cells [173]. NF-*κ*B and p300 were designated as vital nuclear proteins, involved in the modulation of PKC*δ* expression in dopaminergic neurons of *α*-synuclein transgenic mice [181]. Expression of *α*-synuclein at normal level protected neuronal cells against 1-methyl-4-phenylpyridinium (MPP^+^) or rotenone toxicity [182]. Recent* in vivo* and* in vitro* studies provide evidence that *α*-synuclein interacts with histone proteins (p300) and reduces its histone acetyltransferase activity. Subsequent activation of p65 suppresses the binding of general transcription machinery (GTM) to PKC*δ* promoter and represses its gene expression (Figure 3) [179, 181, 183]. PKC*δ* is an oxidative stress kinase and a critical regulator of apoptosis in central nervous system, recognized as downstream substrate for caspase-3. Proteolytic activation of PKC*δ* was reported to be involved in the dopaminergic cell death in cellular models of Parkinson's disease [184–186]. The mechanism through which *α*-synuclein interacts with and disrupts p300 acetyltransferase activity is not yet clear. However, administration of HDAC inhibitors was reported to restore the acetylation state and protect neuronal cells against *α*-synuclein toxicity [187, 188]. In another prime study, it was reported that overexpressed soluble *α*-synuclein forms a complex with antiapoptotic 14-3-3 protein in 54–83-kD complex that makes neuronal cell vulnerable to mitochondrial apoptotic cell death (Figure 3) [155, 189].

**Figure 4:**
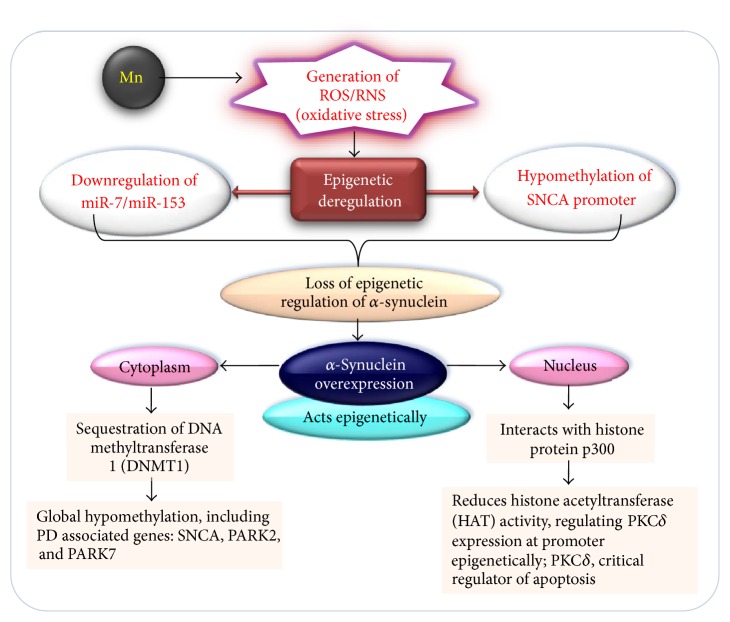
Schematic representing probable involvement of epigenetic deregulation caused by the overexpression of *α*-synuclein in* in vitro* and in* in vivo* cell models of Parkinson's disease. The oxidative stress resulting from Mn exposure has a potential to alter epigenetic regulation of *α*-synuclein via hypomethylation of SNCA gene promoter or through downregulation of miR-7/miR-153. Overexpressed *α*-synuclein acts epigenetically via sequestration of DNA methyltransferase in cytoplasm. This causes the global change in DNA methylation profile of genes associated with PD. *α*-Synuclein inside nucleus regulates the expression of PKC*δ* epigenetically, a proximal regulator of apoptosis. *α*-Synuclein inhibits binding of protein transcriptional machinery to PKC*δ* gene promoter region via binding histone protein p300 and inhibiting its histone acetyltransferase activity.

Recent studies with human postpartum brain sample of PD patients and *α*-synuclein transgenic mice models have reported the association between DNA methyltransferase (DNMT1) and *α*-synuclein, resulting in aberrant cytoplasmic localization of DNMT1 (Figure 4) [155, 189, 190]. Interaction of *α*-synuclein with histone proteins and inhibition of its acetyltransferase activity suggest the direct role of histone proteins in epigenetic regulation (Figure 4).

The sequestration of DNMT1 in cytoplasm results in global DNA hypomethylation including PD associated genes such as* SNCA*,* SEPW1*, and* PRKAR2A* in both human and mice brain samples (Figure 4) [190]. Epigenetic mechanism including DNA methylation regulates the neuronal development and environmental factors were reported to interfere with the regulatory processes at epigenetic level [191, 192]. Developmental manganese exposure in mice was reported to affect neurogenesis in hippocampal dentate gyrus [193]. Global DNA methylation microarray profiling of hippocampal dentate gyrus following manganese exposure revealed hypermethylation of several genes involved in differentiation processes [194]. These findings suggest that deregulation of epigenetic mechanism could be a potential mediator that affects the hippocampal neurogenesis in response to the exposure to manganese. Chronic exposure to manganese has been reported to result in Parkinson's disease in both* in vivo* and* in vitro* cell models. In recent years, environmental epigenetic research has grown very rapidly. Various environmental pollutant induced diseases have been linked with disruption of epigenetic regulation. Manganese exposure could result in neuronal toxicity and neurodegenerative diseases. However, role of manganese induced epigenetic variation in context of neurodegenerative diseases needs to be further investigated. This will help us to develop suitable and effective prognostic and therapeutic approaches.

## 6. Genetic Polymorphism in Epigenetic Susceptibility to Manganese Neurotoxicity

Variation in DNA sequences, epigenetic mechanism, and environmental factors may act synergistically to contribute to the development of neuronal disorders. DNA methyltransferase (DNMT1) maintains the methylation pattern through generations, whereas the* de novo* DNMT (DNMT3A, DNMT3B) introduces the tissue specific methylation marks during the development or in response to environmental factors. Genetic polymorphism in DNMT could be the critical event in epigenetic susceptibility to environmentally induced chronic disease. Recently, a south Indian population was studied for genetic polymorphism in DNMT1, DNMT3A, DNMT3B, and DNMT3L for association with schizophrenia. The genetic variants in DNMT1, rs2114724 and rs2228611, were found to be significantly associated with schizophrenia while* de novo* DNMTs variants, DNMT3B rs2424932, DNMT3B rs156968, and DNMT3L rs2070565, were associated with early age onset of disease [195]. This study had demonstrated the role of genetic polymorphism in DNA methyltransferase and its association with individual methylation level and susceptibility. However, the studies pertaining to the identification of genetic susceptibility factors have been limited and invariantly inconsistent [195]. The individual differences at genetic level determine the response of individual to different environmental stimuli. These differences can be attributed to the variability in response of individuals, even experiencing a similar level of exposure. Epidemiological studies in recent years have reported the positive correlation between the single nucleotide polymorphisms (SNPs) in genes involved in manganese detoxification and differences in individual's susceptibility to manganism [196, 197]. The differences in individual genotypes might be responsible for the variability in blood manganese level of manganese miners experiencing the same exposure environment. It is evident that merely evaluating the blood manganese level in exposed population with individual genotypic difference may not predict the subjects who are more prone to manganese related neurological diseases. In agreement with the above findings, recently research group at our lab have studied the genetic polymorphism in cytochrome P450s, glutathione-S-transferases and NAD(P)H, and quinone oxidoreductase in manganese miners of central Indian population [198]. The finding suggests the association between the cytochrome P450* CYP2D6* polymorphism and susceptibility to chronic manganism [198]. However, the mechanism by which variant of cytochrome P450* CYP2D6* has protective role in manganese susceptibility is not well understood. It might be attributed to perturbation in manganese metabolism or association with other polymorphic alleles of gene that may increase or decrease the enzymatic activity. It is evident from the study that cytochrome P450* CYP2D6* may be considered as a susceptibility biomarker for identifying the miners who are at higher risk for developing manganism. Iron and manganese are supposed to share similar transport and regulatory proteins. It is demonstrated that divalent metal transporter-1 (DMT1) mediates the transport of iron and manganese [199]. Recently, animal studies have found the inverse relationship between the body iron stores and absorption of manganese [34]. It was reported that deficiency of iron resulted in significant increase in manganese absorption and retention in various organs of laboratory animals models [57]. Hepcidin is a regulatory hormone, product of HEP gene that controls the iron absorption in response to total body iron status [200]. The variants of HEP genes, C282Y and H63D, have been reported to be associated with increased uptake of dietary iron [201]. Recent study has found that the individuals with variant of HEP gene had 12% lower blood manganese level in comparison to the wild-type subjects [202]. As discussed earlier that FGF20 is a member of fibroblast growth family that regulates the development and function of central nervous system, it was demonstrated that variation in miR-435 binding site of FGF20 gene, due to single nucleotide polymorphism (SNP), resulted in the FGF20 overexpression [169]. Elevated level of FGF20 has been linked with overexpression of *α*-syn, a characteristic of Parkinson's disease (Figure 3) [169, 196] although the mechanistic insight of the process that leads to *α*-syn overexpression due to elevated FGF20 has not been investigated. However it is evident that the perturbation of epigenetic mechanism (miRNA binding site alteration in FGF20 gene) might be involved in manganese mediated overexpression of *α*-syn, implicated in the development of Parkinson's disease. It may be suggested that the individuals in population having SNP in FGF20 may have altered epigenetic regulation of *α*-syn. Hence these individuals might be predicted at higher risk of developing parkinsonism or other neurodegenerative disorders in response to environment or occupational manganese exposure. Epidemiological studies suggest the definitive link between the genetic polymorphism and epigenetic susceptibility to manganese neurotoxicity. The more comprehensive molecular approaches might give in-depth understanding of the individual genetic differences to association with epigenetic susceptibility in relevance to manganese exposure.

## 7. Conclusion

Epigenetic signatures influenced by manganese exposure need to be studied to gain better understanding of the molecular pathway leading to neuronal cell death and its implication in Parkinson's disease. Most of the findings and interpretation on the mechanism of manganese induced neurotoxicity are based on the high exposure levels of manganese in experimental models, which is considered to induce manganism in humans. This exposure level should not be correlated with chronic low level lifetime exposure in humans. Further studies are needed to investigate whether the molecular pathways that are responsible for manganese induced dopaminergic cell death in humans are similar to those reported in scientific studies using* in vivo/in vitro* cell models. The single nucleotide polymorphism in genes determines the susceptibility level of an individual in response to toxicant exposure. How the epigenetics is linked with genetic susceptibility might be the exciting area of research to be investigated in future. The study of epigenetic mechanism may further elaborate the present knowledge about manganese neurotoxicity. The definitive pathways may be identified which can be used as target for disease progression and therapeutics in relevance to manganese exposure.

## Figures and Tables

**Table 1:** The table shows the major sources and routes of manganese exposure along with their relative potential for exposure to population.

Route of exposure	Source of exposure	Basis for exposure	Relative potential for exposure
Ingestion	Diet	Manganese in breast milk, infant formula, and leafy vegetables, fruit, and fruit juices	Relatively lower for general population but higher prenatal and infant exposure
	Ground water Drinking water Surface water	As a natural constituent of water but elevated level may present at some water source because of pollution sources or bedrock formation	Lower
	Soil	Manganese may present in soil but not a major contributor to exposure	Lower

Inhalation	Ambient air	Higher air concentration nearby industries (mining operations, metal processing plants, etc.) (MMT as additive in gasoline) is cause of concern for residential population	Higher
	Indoor air	Significantly lower but associated with Mn in outdoor dust and the distance from industrial point sources	Relatively lower but implication for childhood exposure
